# Effects of stevia on synaptic plasticity and NADPH oxidase level of CNS in conditions of metabolic disorders caused by fructose

**DOI:** 10.1186/s12906-017-2049-9

**Published:** 2017-12-19

**Authors:** V. A. Chavushyan, K. V. Simonyan, R. M. Simonyan, A. S. Isoyan, G. M. Simonyan, M. A. Babakhanyan, L. E. Hovhannisyian, Kh. H. Nahapetyan, L. G. Avetisyan, M. A. Simonyan

**Affiliations:** 1 Orbeli Institute of Physiology NAS RA, 22 Orbeli Bros Street, 0028 Yerevan, Armenia; 2 H. Buniatian Institute of Biochemistry NAS RA, 5/1 P.Sevag str, 0014 Yerevan, Armenia; 3 Scientific Centre of Artsakh, 8 Tigran Mets str, Stepanakert, Nagorno Karabakh Armenia

**Keywords:** Neuronal plasticity, Neuronal NADPH oxidase, Dietary fructose, Stevia

## Abstract

**Background:**

Excess dietary fructose intake associated with metabolic syndrome and insulin resistance and increased risk of developing type 2 diabetes. Previous animal studies have reported that diabetic animals have significantly impaired behavioural and cognitive functions, pathological synaptic function and impaired expression of glutamate receptors. Correction of the antioxidant status of laboratory rodents largely prevents the development of fructose-induced plurimetabolic changes in the nervous system. We suggest a novel concept of efficiency of Stevia leaves for treatment of central diabetic neuropathy.

**Methods:**

By in vivo extracellular studies induced spike activity of hippocampal neurons during high frequency stimulation of entorhinal cortex, as well as neurons of basolateral amygdala to high-frequency stimulation of the hippocampus effects of *Stevia rebaudiana* Bertoni plant evaluated in synaptic activity in the brain of fructose-enriched diet rats. In the conditions of metabolic disorders caused by fructose, antioxidant activity of *Stevia rebaudiana* was assessed by measuring the NOX activity of the hippocampus, amygdala and spinal cord.

**Results:**

In this study, the characteristic features of the metabolic effects of dietary fructose on synaptic plasticity in hippocampal neurons and basolateral amygdala and the state of the NADPH oxidase (NOX) oxidative system of these brain formations are revealed, as well as the prospects for development of multitarget and polyfunctional phytopreparations (with adaptogenic, antioxidant, antidiabetic, nootropic activity) from native raw material of *Stevia rebaudiana*.

Stevia modulates degree of expressiveness of potentiation/depression (approaches but fails to achieve the norm) by shifting the percentage balance in favor of depressor type of responses during high-frequency stimulation, indicating its adaptogenic role in plasticity of neural networks. Under the action of fructose an increase (3–5 times) in specific quantity of total fraction of NOX isoforms isolated from the central nervous system tissue (amygdala, hippocampus, spinal cord) was revealed. Stevia exhibits an antistress*,* membrane-stabilizing role reducing the level of total fractions of NOX isoforms from central nervous system tissues and regulates NADPH-dependent O_2_^−^ −producing activity.

**Conclusion:**

Generally, in condition of metabolic disorders caused by intensive consumption of dietary fructose Stevia leaves contributes to the control of neuronal synaptic plasticity possibly influencing the conjugated NOX-specific targets.

## Background

Diabetogenic diet causes type 2 diabetes, linked to an increased risk of developing cognitive dysfunction and Alzheimer’s disease (AD) [1–4]. The effects of diabetes on Tau pathology is importany since Tau pathology show a strong relationship to dementia in AD [5]. Hyperglycemia (diabetic pathology) and tau modifications (AD pathology) are tightly correlated and may contribute to the increased incidence of AD, because it has been reported hyperphosphorylated Tau and cleavage in the hippocampus and cortex of type 2 diabetes mice [6]. Furthermore, impairments of brain insulin levels/signaling and glucose metabolism in type 2 diabetes and AD further suggest that an effective treatment strategy for one disorder may be also beneficial in the other [7], obviously, based on the convergence of signaling pathways of insulinotropic and neuroprotective therapies.

High dietary fructose intake promotes the development of pathological characteristics associated with insulin resistance and metabolic syndrome [8, 9] and type 2 diabetes [10]. Systematic review and meta-analysis of the experimental data also confirmed that consumption of high-fructose in beverages caused metabolic syndrome phenotype in rats [11, 12]. New insights into the metabolic consequences of high-fructose diets are conditioned by data regarding the effect of those on the nervous tissue: as fructose-induced insulin resistance in rodents causes a decrease in hippocampal synaptic plasticity [13]. Circulating glucose level is important for cognitive functions, since glucose readily crosses the blood–brain barrier and brain cells use glucose to fuel its activities. Insulin resistance is a major cause of impaired glucose tolerance [14]. Perturbation in insulin signaling pathway, such as insulin resistance impairs hippocampus–dependent memory in humans with type 2 diabetes [15]. Even in individuals without type 2 diabetes a moderate deterioration in glucose tolerance was related to hippocampal atrophy and decreased cognitive function [16]. It has been demonstrated that hippocampus and associated declarative memory function are particularly vulnerable to the effects of type 2 diabetes mellitus [17, 18]. Diet-induced insulin resistanance impairs hippocampal synaptic plasticity and spatial memory in rats [13]. Brain is a target for insulin (non-metabolic functions) and insulin receptors are abundant in the brain, and are highly enriched in olfactory bulb, hypothalamus, hippocampus, cerebellum, amygdala and cerebral cortex [19]. Abundant insulin receptors are found in the limbic system, including the hippocampus and amygdala, regions with reciprocal links and communications and the role of insulin receptors in emotions, learning and memory had been assumed [17]. Amygdala is an essential component of the neuronal circuits involved in emotional response, in general, is thought to attach emotional significance to a stimulus [20]. The most prominent source afferent of basolateral amygdala is the hippocampal formation: the projections are derived from CA1 and entorhinal cortex [21].

The adverse effects of dieary fructose progresses through dysregulation of several signaling factors, and oxidative stress is one of the key molecular mechanisms of fructose-induced metabolic defects [21]. At the same time, the dual role of reactive oxygen species is discussed as cellular messenger molecules for normal synaptic plasticity and as damaging toxic molecules in the age-related impairment of those [22]. It is assumed that K^+^ channels as target proteins can undergo oxidative modulation and their redox sensitivity contributes to altered excitability, progression of neurodegenerative diseases [23]. Features of “two-edged sword” NOX creates a difficulty in therapeutic targeting of this enzyme. Therefore, a therapeutic modulation of NOX activity has to be developed taking in account the disease, the tissue and intracellular localization of NOX isoforms [24], at the same time, provides an overview of the currently available-Nox inhibitors of natural origin [25]. Several plant extracts have been evaluated for their possible therapeutic potential in preventing progression of diseases and as a target for insulin resistance and oxidative stress induced by high-fructose diet [26]. Among the many herbs prevalent in ancient and traditional folk medicine practices, *Stevia rebaudiana* Bertoni is an example, with unique properties, medicinal significance with regard to insulin resistance and diabetes [27]. There is a considerable body of scientific evidence supporting the effectiveness and safety in human health extracts of the leaf of the “sweet herb” Stevia- a natural noncaloric sweetener [28]. The zero-calorie property can also be beneficial to patients suffering from diabetes, as it will not elevate their blood-glucose levels. Steviol glycosides (active compounds of Stevia extracted from leaves) made Stevia an important part of the medicinal world as well as the food and beverage industry [29]. It hase been postulated that *Stevia rebaudiana* Bertoni plant exerts an anti-inflammatory property by inhibiting the activation of NF-κB (nuclear factor 휅-light-chain enhancer of activated B cells) pathway [30], antioxidant properties (the leaves have a greater ability to scavenge free radicals and prevent lipid peroxidation) supported by a large overall proportion of phenols (up to 91 mg/g) [31] and determines the use in folk medicine. In fact, Stevia could have an effect on mechanisms involving free fatty acids, adipocytokines such as TNF훼 and PPAR훾 and serine kinases like JNK and IKK훽, asserted to be responsible in the development of insulin resistance [28]. Besides, Stevioside was also found to reverse scopolamine-induced learning and memory deficits along with attenuation of scopolamine-induced rise in brain acetylcholinesterase activity and brain oxidative stress levels [32]. In cases of metabolic disorders caused by intensive consumption of fructose and correction of those remains insufficiently studied mechanisms of synaptic plasticity. Systematic electrophysiological studies using tetanic high-frequency stimulation (HFS) are in great demand and used to assess the state of the synaptic apparatus [33], in particular, short-term potentiation/depression and the early phase of long-term potentiation/depression that underlie long-term potentiation (LTP) and depression (LTD) formation [34]. Although clinical investigations into LTP in humans are obviously limited, nonetheless they contribute meaningfully to a variety of neurological conditions, including depression, AD, neuropathic and drug dependence [35].

The aim of the present study, in a rat model of metabolic syndrome induced by long-term consumption of dietary fructose, was to evaluate the therapeutic effects of Stevia on: 1) electrophysiological parameters of synaptic plasticity in a entorhinal cortex-hippocampus-amygdala network; 2) the optical spectral characteristics, the specific content, the specific NADPH dependent O_2_^−^ −producing activity of combined fractions of Nox isoforms isolated from the hippocampus, amygdala brain and spinal cord.

## Methods

Historically, rats fed a fructose diet have been used as a model of hepatic dyslipidemia and insulin resistance [36]. High fructose-fed rats have been employed and considered as an experimental model of metabolic syndrome and an alternative nongenetic model for type 2 diabetes [10].

Ecologically pure, high-quality raw material of Stevia (with a high content of biologically active substances), grown in Nagorno-Karabakh was harvested from August to October 2015 [37]. Stevia used in this study was botanically authenticated and voucher specimens were deposited in the Herbarium of Institute of Hydroponics (Experimental Hydroponic Station).

Adult male albino rats weighing 240 ± 10 g were purchased from the experimental center of Orbeli Institute of Physiology. The animals were maintained at 25 ± 2 °C and 12 h light – dark cycle, lights on 07:00–19:00 h. The animals were provided food and water ad libitum. All of the experimental protocols were approved by the Committee of Ethics of the Yerevan State Medical University (YSMU) (Yerevan, Armenia). All animal procedures were carried out in accordance with the European Communities Council Directive 2010/63/UE and the local Animal Care Committee.

Thirty–six rats (240 ± 10 g) were randomly assigned into three groups of 12 each as given below:

Group-C: control rats received water,

Group-F: dietary fructose fed rats (fructose diet contains 20% fructose in drinking water for 9 weeks),

Group-F + St: Stevia treated fructose rats, fed with dietary fructose (20% in drinking water) and treated with Stevia leaf powder (20 mg/kg/day) with food for 3 weeks (start at week 6 until week 9). All animals were fed individually and received a similar amount of Stevia: hungry animals in a separate cage received stevia of the indicated dose (20 mg/kg daily) along with appetizing food (cheese, cottage cheese, meat etc.) and had completely eaten food and transferred to a common cage.

### In vivo electrophysiology

**Fig. 1:**
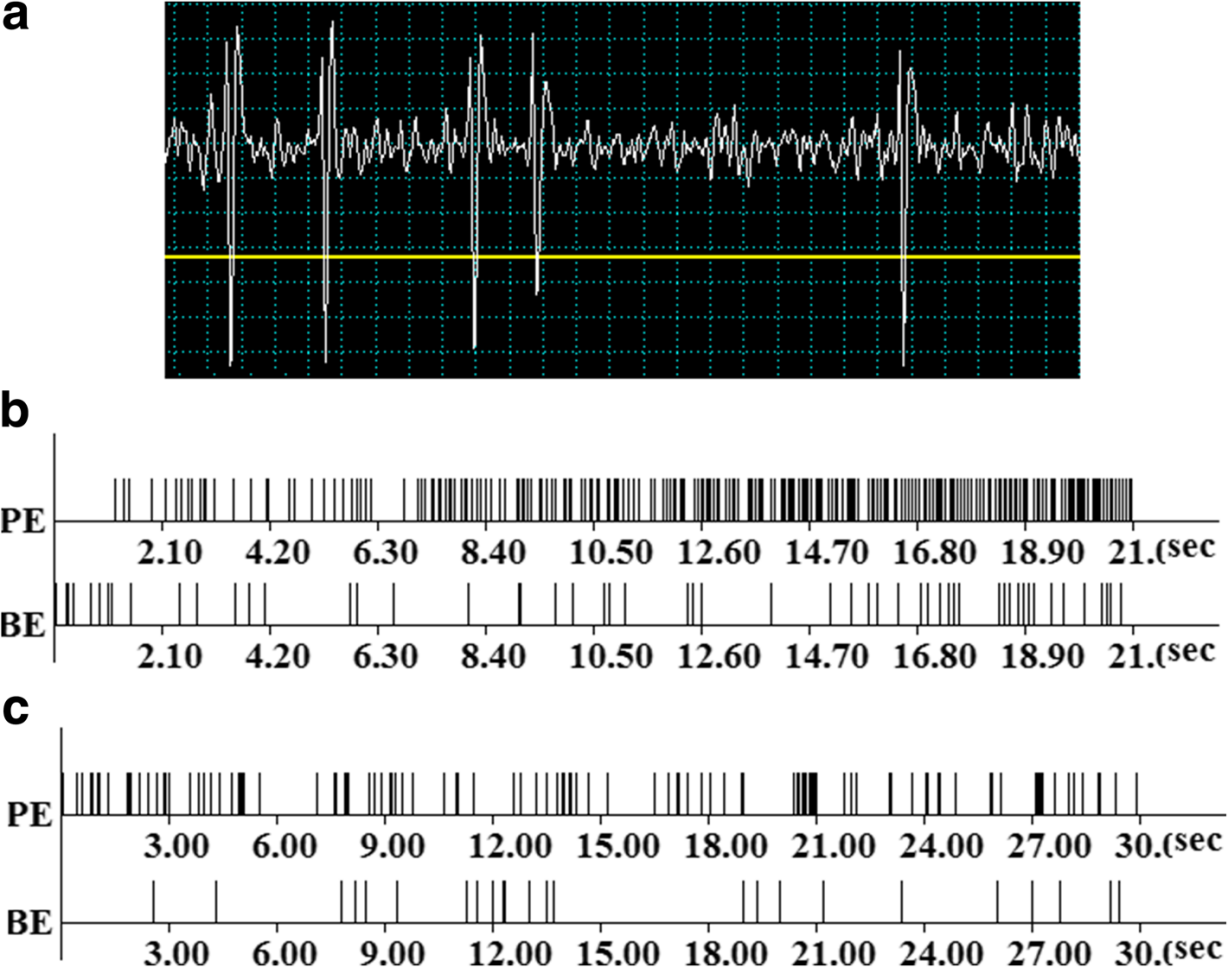
Spike activity of a single neuron of the hippocampus (yellow line- amplitude discriminator) (**a**); prestimulus (BE - before event) and post-stimulus (PE - post event) spike activity in hippocampus (**b**) and amygdala (**c**) in real time 20 and 30 s before stimulation to high frequency stimulation (HFS during 1 s), exhibiting combinations of responses in the form of increased or deceleration frequency of the impulse stream – TD PTP (**b**) and TP PTP (**c**)

**Fig. 2:**
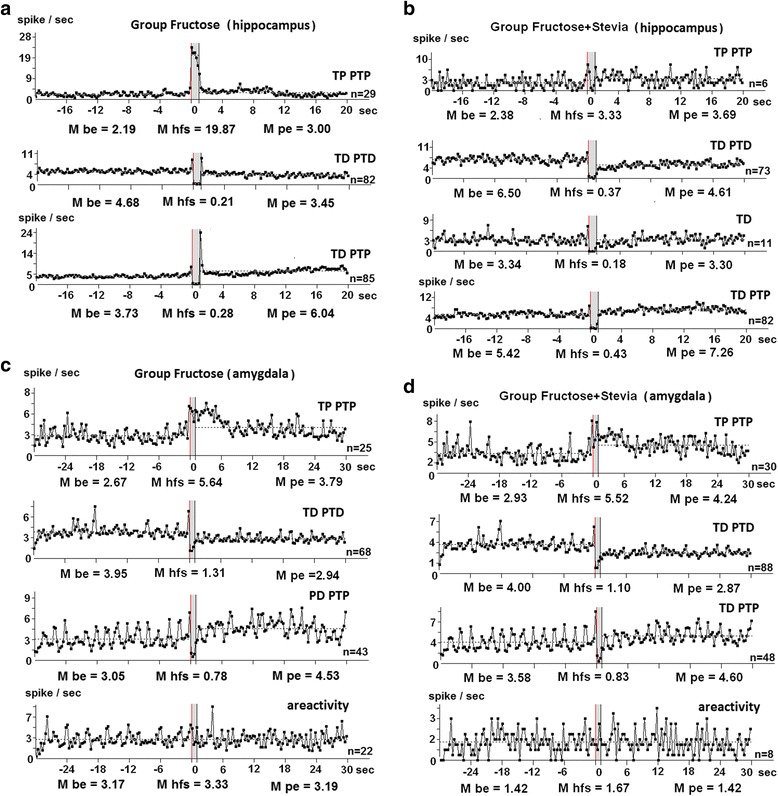
Peristimulus mean frequency diagrams, built on the basis of pre-and post-stimulus manifestations of spike activity of single hippocampal neurons to entorhinal cortex high-frequency stimulation (**a**, **b**) and in single neurons of the amygdala during high-frequency stimulation of the hippocampus (**c**, **d**) in real time and 20 to 30 s before stimulation (Mbe), 20 and 30 s after stimulation (Mpe) and during high-frequency stimulation (Mhfs) exhibiting the specified type of responses (TP PTP, TD PTD, TD PTP) and areactivity in Group-F (**a**, **c**) and Group-F + St (**b**, **d**). Mbe (mean frequency of spikes during time interval before stimulation), Mpe (mean frequency of spikes during time interval after stimulation), Mhfs (mean frequency of spikes during high-frequency stimulation)

After 9 weeks the animals were anesthetized (Urethan 1.1 g/kg), immobilized with 1% ditiline (25 mg/kg i/p), fixed in a stereotaxic head frame and were placed on artificial ventilation. The sample of encephale isole animal was obtained by transection of spinal cord (T2 – T3). The stimulatory electrode was inserted according to stereotaxic coordinates [38] in the ipsilateral entorhinal cortex (AP – 9, L ± 3.5, DV +4.0 mm) and hippocampus (AP −3.0; L ± 2.0; DV +3.5) and a glass recording electrode (1–2 μm tip diameter) filled with 2 M NaCl was repeatedly submerged into the hippocampal and basolateral amygdala fields at coordinates (AP −3.3; L ± 1.5–3.5; DV +3.0–4.0 and AP −3.24; L ± 5.4–5.8; DV +9.5–10.2) for recording spike activity flow of single neurons. High frequency stimulation (HFS) (100 Hz for 1 s) was performed by means of rectangle charge of 0.05 ms duration and 0.10–0.16 mA amplitude. Recording and mathematical analysis of impulse flow (spiking activity) were carried out on the basis of the program (worked by V.S. Kamenetski) providing selection of spikes by amplitude discrimination (Fig. 1 A), which pinpoints spikes and excludes artifacts during HFS, allowing not only posttetanic, but also tetanic activity evalution. The timing (Fig. 1 B, C), frequency and cumulative histograms, as well as a diagram of mean frequency for single neurons and populations of neurons with uniform responses (Fig. 2) were constructed on the basis of analysis of peristimulus spiking. The goal of the analysis was to detect a statistically significant difference in spike activity frequency during prestimulus and poststimulus time intervals, as wel as during HFS. To further evaluate, neuronal units and spike flow of those have significance levels of 0.05, 0.01, 0.001 were selected.

### Isolation of combined fractions of Nox isoforms from the central nervous system

Biochemical studies carried out on brain on the 9 weeks of experiment. The animals were decapitated under deep anesthesia (Pentobarbital 45 mg/kg i.p.) and spinal cord and brain were removed (hippocampus and amygdala were disassociated from the skull). For the production of antibodies during Nox isolation a detergent is used which denatures the enzyme (associated with it) to a certain degree [39]. To prevent this denaturation, a simple method has been developed for isolation of fractions of combined isoforms or single Nox isoforms from cellular and intracellular membranes and nanoparticles of membrane formation-mammalian exosomes, excluding the use of detergent. A combined Nox isoform fraction from mentioned tissues (1 g) was isolated by a specified patented method using the phenomenon of selective complex formation between ferri- or ferrohemoglobin (5–10 μM) with Nox isoforms, located in cell membranes, in intracellular membranes formations, in plasma membranes, as well as in exosomes from blood serum samples [40]. The homogenization of tissues (1 g in 20 ml water) was done by glass homogenizer at 4 °C at 1000 rpm/min for 2 min. Fresh ferrihemoglobin from hemolysate of human erythrocytes was obtained from Hematology Center after prof. Yolyan MH RA. Fresh ferrihemoglobin from hemolysate of human erythrocytes was added to homogenates and incubated at 37 °C and pH 7.4–8 for 1 h and were centrifugated (10,000 rpm/min for 10 min). The supernatant solutions were diluted with water (20–25 times) and were further subjected to ion-exchange chromatography on the cellulose DE-52 column (“Whatman”, England). Combined fraction of the Nox isoforms was eluated from the DE-52 column with 0.1 M potassium phosphate buffer (pH 7.4).

The NADPH-dependent O_2_^−^ −producing activity of Nox isoforms was determined by the nitrotetrasolium blue method. As a unit of NADPH-depending O_2_^−^ −producing activity the amount of Nox isoforms was considered, stimulating the formazan formation by 50%, restoring the nitrotetrasolium blue by superoxide radicals [41]. The NADPH dependent O_2_^−^ −producing activity of NOX isoforms was determined per 1 g of tissue (U/g). Calculated specific content of combined Nox isoforms was determined the optical absorption intensity at 530 nm of 1 ml NOX isolated from 1 g tissue. Experiments were repeated 6 times. The data was statistically evaluated for significance employing Students test. Value of *p* < 0.05 was considered statistically significant. К-70 and K-24 centrifuges “Yanetski” (Germany) and Hitachi-2000 (Japan) spectrophotometer were used.

### Statistical analysis

For statistical evaluation we used t-criteria of Student’s t -test, the reliability of differences of interspike intervals before, after and during HFS. To increase reliability of statistical evaluations, we also used the non-parametric method of verification by application of Wilcoxon two-sample test.

## Results

In vivo electrophysiological extracellular study of activity of single hippocampal neurons evoked by the stimulation of the entorhinal cortex, as well as single neuron activity in amygdala evoked by the stimulation of the hippocampus, and the subsequent analysis of the impulse stream of single neurons revealed the formation of various combinations of responses in the form of increased frequency of the impulse stream - tetanic potentiation (TP) and post-tetanic potentiation (PTP), as well as deceleration of impulse output - tetanic depression (TD) and post-tetanic depression (PTD). Program analysis of peristimulus activity for population of hippocampal neurons (Fig. 2 A, B) and amygdala (Fig. 2 C, D), showing the given type of responses to HFS of entorhinal cortex and hippocampus, respectively, makes it possible to assess the numeric indices of the mean frequency of spike activity before (Mprestimulus), after stimulation (Mpoststimulus) and during tetanization (Mhfs). Thus, according to a detailed analysis, in Group-F tetanic potentiation during HFS in hippocampal neurons with TP PTP is expressed 9 times (19.87: 2.19 spike/s) (Fig. 2 A) (those in Group-C are expressed 4 times). Tetanic depression during HFS in neurons with TD PTD is expressed 22 times (4.68: 0.21 spike/s) in the neurons with TD PTP responses −13.3 times (3.73: 0.28 spike/s) (Fig. 2 A) (those in Group-C are expressed respectively 8.86 and 8.38 times). Thus, in Group-F during HFS have been identified more expressed resposnses compared with Group-C. In Group-F + St tetanic potentiation during HFS in neurons with TP PTP is expressed 1.4 times (2.38: 3.33 spike/s) (Fig. 2 B). Tetanic depression during HFS in neurons with TD PTD is expressed 17.6-fold (6.50: 0.37 spike/s) in the neurons with TD-PTP responses - 11.3 times (5.42: 0.43 spikes/s) (Fig. 2 B). In hippocampal neurons, showing only TD, the expressiveness of thereof was 18.6 (3.34: 0.18 spikes/s) (Fig. 2 B). Thus, in Group-F + St expressiveness of responses to HFS tends to approach those in Group-C.

In Group-C hippocampal neurons had the following balance of types of responses-TD PTD 132 of 294 neurons (44.90%), TD PTP 130 of 294 neurons (44.22%) and TP PTP 32 of 294 neurons (10.88%). In other words, the dominant depressor responses recorded during HFS (tetanic depression was 89.12% (44.90 + 44.22%)) and a small percentage dominance of excitatory responses during post-stimulus time (post-tetanic potentiation was 54.00% (44.22 + 10.88%). In Group-F 190 hippocampal neurons were recorded, of which 23 (12.10%) showed responses in the form of TP PTP, 85 neurons (44.74%) - in the form of TD PTP, 82 neurons (43.16%) - in the form of TD PTD. It can be considered as inhibition dominance during HFS (44.74 + 43.16 = 87.9%) and slight excitation superiority during post-stimulus time (44.74 + 12.10 = 56.8%), which corresponds to those in Group-C. In other words, in Group-F, we have an increase in the percentage of neurons with TP PTP (12.10% instead of 10.88%) responses and a decrease in neurons with TD PTD responses (43.16% instead of 44.90%). Thus, compared to the control in Group-F + St 172 hippocampal neurons were recorded, of which 6 neurons (3.5%) showed responses in the form of TP PTP, 82 neurons (47.7%) - in the form of TD PTP, 73 neurons (42.4%) - in the form of TD PTD, and 11 neurons (6.4%) showed TD. Inhibition dominance is greater during HFS (47.7 + 42.4 + 6.4 = 96.5%) and slight excitation superiority was observed in post-stimulus time (47.7 + 3.5 = 51.2%), which is close to the level of those in Group-F.

According to the detailed analysis on the basis of numerical indices of mean frequency of spike activity before (Mprestimulus), after (Mpoststimulus) stimulation and during tetanization (Mhfs) in neurons of the amygdala Group-F (Fig. 2 C) tetanic potentiation during HFS in neurons with TP PTP is expressed 2.1 times (5.64: 2.67 spikes/s) (those expressed in Group-C 5 times). Tetanic depression during HFS in neurons with TD PTD expressed 3 times (3.95: 1.31 spikes/s), in neurons with TD PTP responses - 3.9 times (3.05: 0.78 spikes/s) (Fig. 2 C) (those in Group-C expressed respectively 3.3 and 5.7 times). In Group-F + St tetanic potentiation during HFS in neurons with TP PTP expressed 1.9 times (5.52: 2.93 spikes/s) (Fig. 2 D). Tetanic depression during HFS in neurons with TD PTD expressed 3.6 times (4: 1.10 spikes/s) in neurons with TD PTP responses - in 4.3 times (3.58: 0.83 spikes/s) (Fig. 2 D).

Thus, under conditions of long-term consumption of dietary fructose a key role in the plasticity of entorhinal-hippocampus-amygdala cortex networks have an increase in the hippocampus and decrease in the amygdala the expressiveness of depression and excitation during HFS. Under conditions of combined consumption of dietary fructose and stevia was recorded a) in hippocampal neurons tended to be norm levels of increased frequency (excitation) and decreased frequency (inhibition) of spikes during HFS and post-stimulus time; b) in neurons of amygdala close to norm levels of depression during HFS.

In Group-F 158 amygdala neurons were recorded, of which 25 (15.8%) during HFS of hippocampus showed responses in the form of TP PTP, 43 neurons (27.2%) – in the form of TD PTP, 68 neurons (43%) as TD PTD, 22 neurons (13.9%) showed no response. It can be considered as the dominance of inhibition during HFS (43 + 27.2 = 70.2%) and an equivalent balance of post-tetanic potentiation (15.8 + 27.2 = 43%) and post-tetanic depression (43%). In Group-F + St 174 amygdala neurons were recorded, of which 30 neurons (17.24%) showed responses in the form of TP PTP, 48 neurons (27.59%) - in the form of TD PTP, 88 neurons (50.57%) - in the form of TD PTD and 8 neurons (4.6%) showed areactivity. It can be considered as a dominance of inhibition during HFS (50.57 + 27.59 = 78.16%), and a shift in the balance of posttetanic responses in favor of inhibitory (posttetanic depression was 50.57%, and the posttetanic potentiation - 44.83%). In Group-C 168 neurons were recorded and showed the following types of responses: TP PTP (25%, 42 neurons), TD PTP (25%, 42 neurons), TD PTD (35.7%, 60 neurons). Thus, percentage ratio of inhibition during HFS is 60.7% (35.7 + 25%), which is slightly prevailing over tetanic potentiation, while in posttetanic responses dominates post-tetanic potentiation (25 + 25 = 50%) (post-tetanic depression is 35.7%). Areactive units were 14.3% (24 neurons). Thus, compared to the control under the influence of fructose in amygdala neurons a deviation towards the increase in ratio of inhibitory reactions was revealed. Under the influence of Stevia in hippocampal and amygdaloid neurons during tetanization and post-stimulus period the prevalence of inhibitory responses was recorded.

Thus, in condition of metabolic disorders caused by long-term consumption of dietary fructose have been detected electrophysiological indices of abnormal synaptic activity of entorhinal cortex-hippocampus-amygdala neural networks with typical disturbances of balance and intensity of excitatory/inhibitory evoked responses. These abnormalities apparently are a result of insulin signaling system since the insulin is an important neuromodulator and affects electrophysiological properties of neurons [42].

## Discussion

Neuronal insulin receptors play important roles in neuronal survival, neuronal circuitry formation and synaptic plasticity [43], and during neuronal maturation regulate postsynaptic neurotransmitter receptor trafficking [44]. Abnormal glutamate receptors [45], decrease in amplitude and long-term delay in evoked potential components [46], preferred affectation of postsynaptic AMPA receptors [47] are characteristic indicators of the central nervous system of diabetic animals. Our data correlate with the shifting of ratio of TP PTP, TD PTD, TD PTP responses in conditions of intensive consumption of fructose. Moreover, assessing the short-term plasticity (1 s during tetanization and up to 20–30 s for the post-stimulus time interval of recording) in this study in a model of type 2 diabetes was revealed: 1) increased TP and TD responses (that may result from activation of “silent” AMPA receptors [48], or their conformation [49] or sensitization [50]); 2) decrease in peristimulus spiking (apparently as a result of an adaptive decrease in the functional activity of NMDA receptors). We estimate these data as a manifestation of the CNS plasticity in conditions of intensive consumption of fructose. It is important to remember that activity-dependent synaptic plasticity in insulin-resistant rats manifested itself in decrease in LTP in CA3-CA1 synapses, while LTD remained unaffected [51], which is consistent with our data on the lowest vulnerability of neurons with depressor responses, as well as the adaptive role of predominant inhibition under the influence of Stevia. Thus, under conditions of combined consumption of fructose and Stevia, we found close to norm indicators of background activity, as well as percentage balance and intensity of excitatory/depressor reactions during high-frequency stimulation and for post-stimulus time, indicating the preservation of the plastic properties of the cortex-hippocampus-amygdala network.

Long-term potentiation is a form of synaptic plasticity and an important electrophysiological parameter and was found to have significant effect on learning and memory processes [52]. According to many studies, defects of long-term potentiation in the hippocampus of diabetic animals might arise from dysfunction of the N-methyl-D-aspartate (NMDA) subtype of glutamate receptors. From an electrophysiological point of view, reduction in the amplitude and long delays of components of evoked potential has been described in the central nervous system of adult patients with type 1 and 2 diabetes mellitus. Employing this experimental approach of paired-pulse facilitation, Biessels et al. (2002) found that paired-pulse facilitation was not modulated in a model of streptozotocin-induced type 1 diabetes, reinforcing that chronic diabetes in rats does not interfere with presynaptic properties but rather with postsynaptic mechanisms (i.e. regulation of AMPA receptors) contributing to maintenance of long-term potentiation [47]. Moreover, there is growing evidence that the expression of synaptic depression is mediated by modifications of postsynaptic currents produced by AMPA subtypes of glutamate receptors [53]. During high-frequency stimulation, transient hyperactivity of NMDA receptors is responsible for appearance of long-term potentiation in numerous brain pathways known to be involved in learning and memory processes. Consequently, it is arguable that deficits in synaptic plasticity in the early stages of diabetes might reflect pre-existing expression of synaptic potentiation (the so-called occlusion effects) rather than increased sensitivity to excitotoxicity [54]. In this regard, diabetes should lead to stimulation of “silent” synapses in the hippocampus. Such changes of responses might be caused by an increase in AMPA receptor conformations. Our previous studies, in the same experimental model of long-term consumption of fructose in hippocampal and amygdaloid neurons (during HFS of entorhinal cortex and hippocampus, respectively), revealed a predominant activation of excitatory neurotransmission during single intramuscular systemic injection of therapeutic dose of stevioside – the main pharmacologically active component in leaves of the Stevia plant [55]. It is interesting to note that in cases where proteins such as phosphodiesterase 1 (PDE1), responsible for the cyclic adenosine monophosphate (cAMP) degradation concomitant with stevioside treatments is downregulated, cAMP (essential in amplifying insulin secretions physiologically induced by glucose) will be increased, suggesting stevioside’s ability to holistically amplify the expressions of glucose-responsive genes [30], which presumably can extrapolate the possibility of activation of second messengers such as cAMP to highlight the neurotransmitters. Data about the impact of stevioside on neurotransmitter systems are very scarce. It is known that treatment of hippocampal cultures with *Stevia extract* induced biochemical markers typical for LTP (pCREB: activated cAMP response element binding protein; pMAPK: activated mitogen-activated protein kinase; surface expression of the AMPA Receptor Subunit GluR1 (https://www.google.com/patents/WO2009071277A1). It has also been found in accordance with the invention that Stevia extract has the ability to inhibit glycine reuptake by inhibiting the glycine transporter GlyT1 (https://www.google.ch/patents/US20110038957). GlyT1 is the only sodium chloride-dependent glycine transporter in the forebrain, where it is co-expressed with the NMDA receptor. At this site, GlyT1 is thought to be responsible for controlling extracellular levels of glycine at the synapse [56], resulting in modulation of NMDA receptor function. Such data highlight the potential usefulness of compounds, which can enhance NMDA receptor synaptic function by elevating extracellular levels of glycine in the local microenvironment of synaptic NMDA receptors, for maintaining physiological cognitive functions. Stevia extracts allow accumulation of glycine in the vicinity of the NMDA receptor, thus activating it and ultimately resulting in the induction of LTP. However, long-term increase in extrasynaptic glycine concentration may cause more diffuse and prolonged tonic inhibition (carried out by extrasynaptic receptors) againsta phasic inhibition [57]. In the regulation of neuronal excitability by Stevia, the total depression caused by extrasynaptic increase in glycine may exceed the value of phasic transmission, which forms the balance of potentiation and depression. Modulation of background activity, preservation of the level of general activity, selective suppression of certain input routes, plastic changes in inhibitory synapses through transcriptional regulation and expression of receptors ultimately results in the consolidation of spatial coding in neural networks of our animals in F + Stevia group.

In the clinical practice glibenclamide is widely used in the treatment of diabetes mellitus type 2. During the last decade, glibenclamide has received renewed attention due to its pleiotropic protective effects in acute CNS injury (involving the sulfonylurea receptor 1 (Sur1)) in acute pathological conditions of the central nervous system, providing new opportunities for its use [58]. Our previous studies (as a positive control) in conditions of prolonged consumption of dietetic fructose, Glibenclamide (a sulfonylurease drug used to treat type 2 diabetes) modulates the plasticity of the entorhinal cortex-hippocampus-amygdala network by shifting the percentage balance in favor of depressor types of responses during HFS and an increase in their intensity. On the model of intensive fructose intake by evaluating the electrophysiological parameters of synaptic activity in real-time dynamics after the injection of a single therapeutic dose of Glibenclamide, we have also revealed the activation of predominantly excitatory reactions in the hippocampal and amygdala neurons. Electrophysiological indices of directionality of the effects of stevioside and Glibenclamide in the CNS are identical [59].

We can assume that under impaired glucose metabolism conditions stevia adapts neuronal brain network by stablishment of depression/or strengthening of depression mediated by activation of excitatory neurotransmitters. Data on the short-term activation and long-term depressor effects were in favor in the functioning of Stevia as an allosteric modulator, which is consistent with the concept of molecular and cellular factors controlling signal transduction via transferred allosteric modulator proteins, a key one being G-protein-coupled receptor for natural substrates [60]. At the systemic level, allosteric modulation of Stevia provides for neuroprotection by long-term changes of allosteric ligands, which in turn leads to an increase in neurotrophic factors.This intriguing possibility requires further investigation.

### Biochemistry

**Fig. 3:**
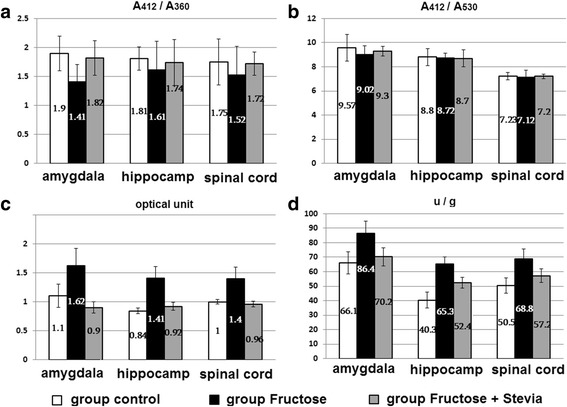
Optical spectral absorbance indices of A_412_/A_360_ (**a**) and A_412_/A_530_ (**b**) of total fraction of Nox isoforms, isolated from the amygdala, hippocampus and spinal cord of rats of these groups (M ± m; *n* = 6; *p* < 0,05). (**c**) - relative optical absorbance of total fractions of Nox isoforms from the amygdala, hippocampus, spinal cord (per optical units), and (**d**) – calculated ratio/proportion of the NADPH- dependent O_2_^−^ −producing activity of total fraction of Nox isoforms (U/g tissue) in these groups (M ± m; n = 6; *p* < 0,05)

Selective complex formation between Hb and Nox isoforms was proved in three independent ways: formation of S-nitrosohemoglobin, capture of traces of Hb in suprol (superoxide-producing lipoprotein of serum [39]) and complex deposition on cellulose DE-52 [61–63]. Hb-induced releasing of Nox isoforms of total fraction from heterogeneous phase (from biomembranes) in soluble phase occurs through this complex formation. A method of producing highly pure total fractions of Nox isoforms has been developed using this process without using denaturing agents [64, 65]. The process of Hb-induced releasing of total fractions of Nox isoforms inhibited by antioxidant compounds, which have a membrane-stabilizing effect inhibiting process of this releasing [66–68]. Optical absorption spectra of combined fractions of Nox isoforms (Nox family: 22 phox, 91 gphox, 40 phox, 47 phox, 67 phox), isolated from rat amygdala, hippocampus and spinal cord of Group-C, Group-F and Group-F + St are shown in Fig. 3. Fractions of isoforms of separate or combined Nox are acid haemoproteins and, as have already mentioned, was eluted from the DE-52 column with 0.1 M potassium phosphate buffer. Given absorption spectra of fractions of combined and separate Nox (using Sephadex G-100) in Group-C, Group-F and Group-F + St do not differ in form and maximum optical absorption (figures are similar and not presented), but they differ in optical spectral indices (A_412_/A_360_ and A_412_/A_530_), as shown in Fig. 3 A, B. Optical spectral characteristics and optical absorption peak of combined fractions of Nox isoforms in Group-C, Group-F and Group-F + St from the amygdala, hippocampus and spinal cord in oxidized and reduced state is consistent with the indices of total fraction of Nox isoforms, isolated in this way from other tissues of rats and in the brain [41].In the oxidized form this cyt b 558 has maximal absorption at 560 nm (α-absorption), 530 nm (β- absorption), 412 nm (γ absorption), and absorption at 350–360 nm. Reduction of Nox by sodium dithionite resulted in the appearance of characteristic optical spectral absorbances at 558 nm, 525 nm, 418 nm. However, the values of the optical spectral indices (the ratio of A_412_/A_360_ and A_412_/A_530_) given in Nox Group-C, Group-F and Group-F + St are slightly differ from those of Nox from other tissues, in particular from the lungs of rats by increasing the density of optical absorption at 350–360 nm (Fig. 3 A, B) as a result of increase in the content of flavin adenine dinucleotide (FAD) in Nox [69]. As shown in Table 1, compared with indices of Group-C, in Group-F a decrease in the optical spectral index (A_412_/A_360_ and A_412_/A_530_) of total fraction of Nox isoforms from cellular components of the amygdala, hippocampus and spinal cord was observed. On the other hand, under action of stevia in Group-F + St these indices approach the Group-C indices without further changes in optical Nox indices. Free or protein-bound FAD Nox has maximum absorbance at 350–360 nm. It is known that Nox isoforms are FAD-containing hemoproteins – cytochrome b558, and, thus, FAD acts as an activator of Nox and as an electron source [70, 71]. Changes in the estimated content and NADPH-dependent O_2_^−^ −producing activity of the combined fractions of Nox isoforms in Group-C, Group-F and Group-F + St also occur inappropriately (Fig. 3 C, D). Compared with indices of Group-C in Group-F a significant increase in the level of cleaved Nox isoforms and their NADPH-dependent O_2_^−^ producing activity was observed. In Group-F + St the data approach the Group-C indices (Fig. 3 C, D). Thus, we can argue that stevia plays antistressor, membrane stabilizing effect upon the decrease of the degree of released of total Nox isoforms from amygdala, hippocampus and spinal cord, correspondingly was decrease the membrane destabilizing influenc of hemoglobin. In Group-F an increase in the share of total content of isolated fractions of Nox isoforms of CNS tissues is associated with increase of lipid peroxidation in these tissues. These radicals cause tissue damage by an increase the lipid peroxidation and then, Nox release level upon increase the degree of selective complex formation between hemoglobin and Nox located in biomembranes [66].

Increase in NADPH-dependent O_2_^−^ − producing activity of released total fraction of Nox isoforms of amygdala, hippocampus and spinal cord in Group-F compared with the Nox Group-C indices associated with an increased level of FAD (increase in density of maximum optical absorption at 350–360 nm, or decrease of optical spectral index (A_412_/A_360_) of the fraction of Nox isoforms were take plase. In fact, FAD is an activator [72] and for Nox amygdala, hippocampus and spinal cord. Another factor for activation of presented Nox can be increase of the Nox phosphorylation degree, released from the mentioned tissues, because Nox phosphorylation level in the CNS is increased [66]. As a result of this, in Group-F was observed an increase in (3–5 times) NADPH-dependent O_2_^−^ − producing activity of the total fraction of Nox isoforms released from CNS.

Previously, we observed a significant increase after 6 (5,97 ± 0,3 mmol/l *P* = 0,03) and 9 (7,53 ± 0,2 mmol/l *P* = 0.001) weeks from the start day of fructose drinking (fructose was administered as solution in drinking) compared to baseline mean blood glucose values (5,07 ± 0,2 mmol/l) in Group-F. In Group-F + St data obtained from animals suggested that glucose level increased in 6th week (6,45 ± 0,3 mmol/l *P* = 0.004), in which the use of stevia along with fructose during experimental weeks (6–9) decreased glucose level (5,42 ± 0,09 mmol/l *P* = 0.13), not significantly distinguishable from starting mean level (5,13 ± 0,15 mmol/l) in this group [72]. It is shown that NADPH oxidase inhibition prevents beta cell dysfunction [73], and Stevia extracts could mimic insulin effects by modulating PI3K/Akt pathway [74], and anti-hyperglycemic effects of Stevia we attributed to its antioxidant properties. Moreover, in the same paper [72], we have shown that Stevia inhibits releasing of Nox isoform from cellular and intracellular membranes of spleen, lung, liver, erythrocyte membranes of blood serum of rats on high*-*fructose diet due to its high SOD-mimetic activity (20 units/mg), thereby showing membrane-stabilizing effect associated with protection against the damaging effects of superoxide and hydroxyl radicals – active stimulators of tissue lipid peroxidation and oxidative stress. In addition, Shivanna et al. [31] a significant decrease (about 30%) in peroxidation in the livers of Stevia-pre-fed rats was observed compared to those of their control groups. Dietic fructose has been shown to stimulate in rodent [75] and in human [76, 77] de novo lipogenesis, *e*levated levels of plasma triglycerides*,* increase in hepatic lipids and visceral obesity. Treatment with antioxidants prevented the development of oxidative stress phenomena associated with compensatory hepatic metabolic mechanisms [78].

In the present study, in a model of fructose-induced diabetic stress, Stevia shows membrane-stabilizing effect on central nervous system cells, as well as exhibits SOD-mimetic activity. It should be noted that the activities of superoxide dismutase and catalase, two major enzymes involved in brain detoxification, are reduced in the streptozotocin-diabetic model, whereas brain superoxide dismutase is increased in type 2 diabetic animals [64]. Recent evidence suggest that deregulation in the production of reactive oxidative species (ROS) by fructose diet leads to DNA damage, lipid peroxidation and aberrant post-translational modification of proteins, which may directly impact localization of proteins and vesicles across the soma, dendrites and axon of hippocampal neurons [79]. A subtle balance of ROS is a deterioration of cellular mechanisms of hippocampal plasticity underlying learning and memory [80]. In other words, the authors have suggested amendments to traditional view that brain ROS are solely deleterious by demonstrating that controlled ROS chemistry is needed for maintaining specific cell populations, in particular, the hippocampus [81]. Membrane proteins, gp91^phox^ and p22^phox^ and cytosolic proteins, p40^phox^, p47 ^phox^, p67^phox^ have a specific function and were localized in neuronal cell components as well as in synaptic sites of mouse hippocampal dendrites [82], which may play a definite role in recorded synaptic and spike activity in hippocampal and amygdaloid neurons. In rodents, high-fructose diet decreases the phosphorylation level of insulin signaling pathway in hippocampus and cortex and contributes to cognitive impairment [83]. Fructose diet can increase neuronal uptake of fructose: fructose transporter demonstrates increased expression of mRNA, accompanied by an increase in GLUT5 level in the hippocampus. It is known that high-energy diet may reduce the memory triggered by an onset of central insulin resistance [84]. The inclusion of *Stevia* leaves in the diet has been associated with antihyperglycemic, insulinotropic, glucagonostatic, hypotensive, anti-inflammatory and immunostimulatory responses [85]. Considering that progression of insulin resistance and metabolic syndrome associated with reactive oxygen and tumor necrosis factor alpha (TNF-alpha) a potential activator of c-Jun NH(2)-terminal kinase (JNK) [86], as well as potential role of stevia in abrogating insulin resistance through regulation of adipocytokines such as TNFα and PPARγ (peroxisome proliferator activator receptor γ), JNK and IKK*β* (inhibitor of nuclear factor *к*B kinase *β*) [28], we suggest the involvement of these molecular mechanisms in the formation of protective effects of stevia in the CNS against the ravages of excessive fructose consumption. Plant extracts have anticholinesterase activity and can be considered as sources of neuroprotective phytomolecules in alternative therapies and a positive correlation was observed between total phenolic content [87].

## Conclusion

Under conditions of intense consumption of dietary fructose, stevia inhibits releasing of total fractions of Nox isoforms from CNS tissues and regulate the NADPH-dependent O_2_^−^ −producing activity of Nox by SOD-mimetic activity. Stevia showed membrane-stabilizing effect upon decrease the release of the isoforms of Nox from CNS. Electrophysiological parameters of hippocampal and amygdala neurons in condition of dietary fructose consumption revealed and point to the key role of depression during HFS and a practically equivalent balance of excitation/depression during post-stimulus time to generate the optimal activity in the entorhinal cortex-hippocampus-amygdala networks, associated with memory processes. Stevia reduces level of expressiveness of potentiation/depression during HFS and post-stimulus time in the hippocampus and increases the level of expressiveness of depression during HFS and post-stimulus time (becomes closer to the norm) by shifting the percentage balance in favor of depressor types of neuronal responses during HFS that indicates plasticity modulation of networks. The results provide some contribution to understanding the role of Nox-specific targets controlling brain plasticity treated with Stevia leaves in condition of metabolic disorders caused by excessive dietary fructose consumption.
